# A plan to transform primary and community care at Catalonia based on a process improvement methodology

**DOI:** 10.1017/S1463423624000604

**Published:** 2024-12-02

**Authors:** Marc Sales Coll, Sara Manjón, Jeroni Salabert, Clara Pladevall, Daniel Algar, Alba Benaque, Júlia Soler, Anna Ochoa de Echagüen Aguilar

**Affiliations:** 1 Department of Business Organization, Management and Product Design, University of Girona, Girona, Spain; 2 Essentia Health Management, Barcelona, Spain; 3 Servei Català de la Salut, Barcelona, Spain

## Abstract

**Aim:**

This article outlines the implementation and deployment strategy defined by the Catalan healthcare system which sought to promote a plan to strengthen and transform primary care in order to provide high-quality healthcare services whilst making an optimal use of resources across the Catalan region.

**Background:**

Following the COVID-19 pandemic, the Catalan healthcare system initiated a plan to enhance primary care services. The Lean methodology has been used extensively in other sectors for process improvement. More recently, it has been adopted in large hospitals, showing good results, so Lean was selected as the most appropriate method to achieve this project’s goal. The Process Office of the Catalan Health System, which is made up of experts in Lean methodology, has been involved in defining and deploying the strategy to all 374 Primary Care Teams (PCTs) within the Catalan healthcare system.

**Methods:**

The deployment strategy was executed in four phases (each consisting of a number of sessions): (1) explaining the project’s goals and training the professionals in the methodology; (2) assessing the current status regarding the processes with the various PCTs; (3) identifying and implementing improvement projects; and (4) defining key performance indicators to monitor the impact of the projects.

**Findings:**

As a conclusion, this project has allowed to successfully define and implement a standard at a strategic and deployment level of a project for primary care improvement that may be replicated in other regions. The key elements to ensure success have been the following: leadership, implementation of an improvement methodology, standardization of sessions, and involvement and training of front-line professionals.

## Introduction

### Status of primary care in Catalonia

The trend regarding demand for primary and community care was seriously impacted by the COVID-19 pandemic in 2020, where healthcare systems had to cope with the challenge by adapting to the new situation and prioritizing COVID-19 patients. In addition, in order to ensure an adequate response in the future and get on top of the backlog created during the lockdown, primary care experienced an increase in activity and the workloads of professionals were exacerbated.

According to the World Health Organization (2021), primary and community care is key for making healthcare systems more resilient during crisis, more proactive in the detection of epidemic signs, and more prepared to respond to sudden increases in service demand. Aside from the fact that it has become clear how important is the PACC structure during a pandemic, it is also clear that, with the ageing population and the increased prevalence of chronic diseases, there is a clear trend towards an increased demand for this service. For these reasons, it is also important to introduce changes that allow to optimize the management and organization of primary care processes in order to address this.

The Catalan Healthcare Service (CatSalut) is the public body linked to the Catalan Ministry of Health (Departament de Salut de la Generalitat de Catalunya), which ensures the provision of comprehensive, quality public healthcare to all residents of Catalonia through an adequate management of the resources to meet the needs of the population. In 2020, following the onset of the pandemic, the ‘Pla d’Enfortiment i Transformació de l’Atenció Primària i Comunitària’ (PEiTAPiC, literally translated as Strengthening and Transformation Plan for Primary and Community Care) was promoted in order to provide citizens with top-quality healthcare and to do so with a rational use of resources. The PEiTAPiC set out very specific goals and actions for improving accessibility, decision-making, user experience, work environment, and integration between levels across healthcare systems.

### A change is needed in the management and organization of healthcare

It is clear that health processes can be improved and, although healthcare is one of the sectors with the greatest concentration of professional talent, training, and motivation, the introduction of new methodologies aimed at operational excellence and value generation is challenging and sometimes can be ineffective. The day-to-day involvement and demand make it difficult for managers and professionals to devote time to think about innovative solutions and/or rearrangements. In addition, healthcare services involve a number of groups (doctors, nurses, care technicians, nursing assistants, administration personnel, etc.), and there is room for improvement in their joint efforts to provide value, which evidences areas of inefficiency (Martínez Ibáñez *et al*., 2020).

The many aspects that do not add value to the care process (delays, interruptions, unnecessary movements, duplication of tests, and many other inefficiencies) end up causing great burnout to the professionals, who strive to give the best care, and at the same time a poor perception of the service by the user (Martínez Ibáñez *et al*., 2020; Hallam & Contreras, 2018; Godley & Jenkins, 2019; Simons *et al*., 2017).

Fortunately, there is a growing tendency to focus on the most important aspect of any healthcare system: THE PATIENT. This entails and requires an organizational change, from a vertical to a cross-sectional view from the user perspective, focusing more on care processes and with professionals working as a coordinated and multidisciplinary team.

### Lean methodology for improving care processes

In recent years, new management methodologies and approaches are being introduced to organizations, remarkably improving the work environment and the perception by the user of the service received. When we look at the industrial sector, we see that Lean Management has been used for over 50 years to improve the efficiency of organizations with a very active involvement by the employees (Liker, 2004). This methodology has possibly been the one that has had the greatest impact on improving industrial processes, although there are others that are mostly inspired by the same principle: identifying value from the customer’s point of view. In the healthcare sector, process optimization tools such as the ones provided by the Lean methodology have been implemented for 20 years (Coughlan & Coghlan, 2002; Bonome *et al*., 2016; Tlapa *et al*., 2020), but it is only in the last decade that they have been used in a standardized way in the US (Henrique *et al*., 2016; Abdelhadi, 2015; Wickramasinghe *et al*., 2014; Andersen & Røvik, 2015; Toussaint, 2009; Bruno, 2017; Sisler *et al*., 2017; Cohen, 2018), Canada (Baker, 2014), and some European countries (Visintin *et al*., 2017; Perona *et al*., 2016; D’Andreamatteo *et al*., 2015; Hicks *et al*., 2015; *Going Lean in Health Care*, 2005; Sales & De Castro, 2021). Based on this evidence, the PEiTAPiC deployment proposed by CatSalut using the Lean methodology was suggested to identify value for the customer (the primary care user) and optimize the processes that have a direct impact on the care process.

### Scope of action

**Figure 1.**
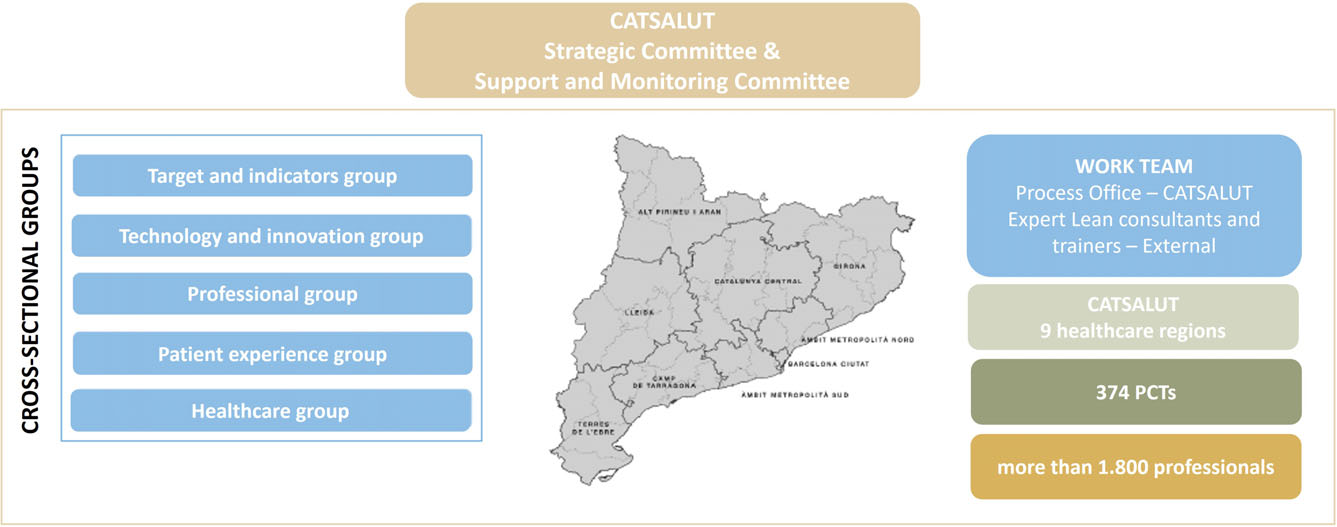
Working team, scope, and governance of the PEiTAPiC project.

The scope of action comprised 374 PCTs, which involved working with around 1,800 professionals from a number of disciplines within a PCT: medicine, nursing, administration, and site management (Figure 1).

### General aim

Defining the strategy to ensure the implementation and operationalization of the PEiTAPiC by means of a consistent deployment across the region, reaching each and every team within the system in a consistent and involved manner, ensuring the appropriate implementation and long-term continuity.

## Methodology

The methodology implemented for the PEiTAPiC is built on several key pillars, each essential for ensuring the effective transformation of primary care across Catalonia. These pillars form the foundation of our approach, guiding the establishment of the work team, governance structures, and the phased deployment strategy that underpins the entire initiative.

### Work team

- A team of professionals from the process office at CatSalut’s Care Area, consisting of six process engineers distributed around the territory, three senior process engineers, and the project director. This team is responsible for the coordination and deployment of the project following a management model based on the application of the Lean methodology, involving all kind of primary care professionals throughout the process.
- A team of external consultants with expertise in Lean methodology. Their role is to collaborate closely with the process team to support and coordinate the entire deployment of the project, ensuring that the Lean principles are applied effectively while working with the primary care professionals.
- A team of external trainers responsible for providing foundational training on Lean methodology to the primary care professionals. This training is delivered in parallel with the project deployment, equipping these professionals with the necessary knowledge and tools to understand the Lean methodology and enabling them to actively participate in the project.
- Five cross-sectional groups made up of professionals with expertise in various areas of knowledge. These experts develop projects at the system level: target and indicator group, technology and innovation group, professional group, patient experience group, and healthcare group.

### Governance

A **governance model** having two committees in parallel was established: the Strategic Committee and the Support and Monitoring Committee, led by the project leader, and supposed to hold a monthly and a weekly meeting, respectively.

The Strategic Committee was represented by several cross-sectional groups, the project leader, the three coordinating engineers and the external consultants. This committee was set up with the aim of ensuring the adequacy and feasibility of the projects suggested by the process teams, as well as to determine strategic projects for CatSalut that were to be deployed across the region.

The Support and Monitoring Committee, consisting of the six process engineers from CatSalut’s regional team, the three coordinating engineers, the project leader and the team of external consultants, conducted the operational monitoring of the projects and services.

### Deployment

**Figure 2.**
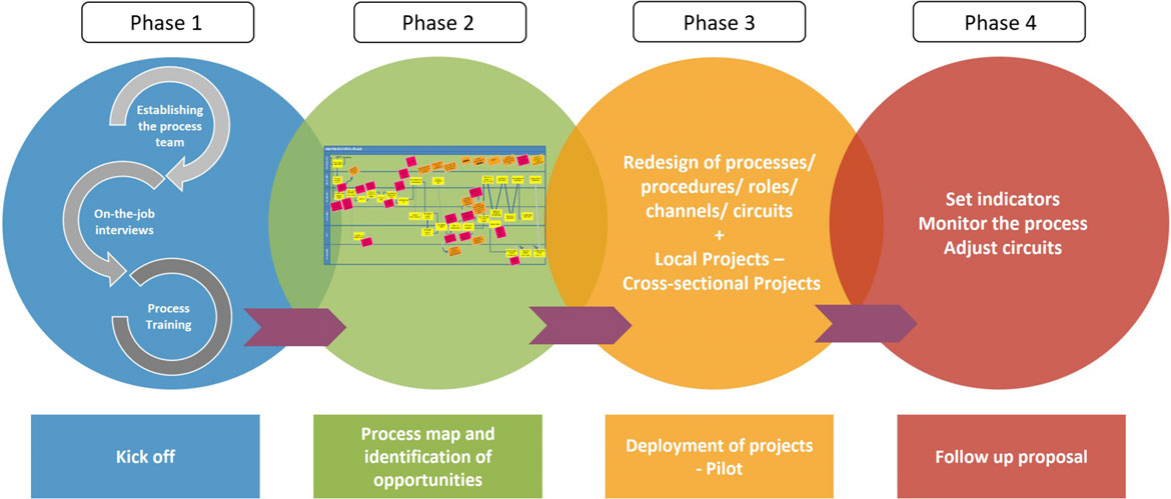
Phases for the implementation of the Transformation Plan.

In order to address the wide scope of the project and implement a methodology for improving processes in a standardized way across the region in a consistent manner, the suggested roadmap for the deployment model consisted of four phases (see Figure 2) that initially were developed according to the early proposal in a set of eight standardized working sessions.

The PEiTAPiC plan was presented, and the deployment model was suggested to CatSalut’s regional directive structures (Healthcare Regions) and the directive structures of primary care providers.

#### PHASE 1 – Kick-off

The first phase consisted of:

*1. Establishing the process team and starting the project*

In order to create synergies between the nearest PCTs and the relevant healthcare region, we created multidisciplinary teams, which included professionals with a global view of the processes to be evaluated.

Each PCT appointed a minimum of three professionals (medicine, nursing, and administration) to create the process team. Once the process team had been created, the first ‘kick-off’ session was held to start the project for each PCT and to suggest a schedule for upcoming sessions. The site director, the professionals appointed to be part of the process team, and the directors of the organizations were involved.

*2. Evaluating the starting point*

To put into context what was the starting point of the PCTs, an early exploration of the needs was conducted with the aim of identifying key points and areas for improvement. This first exploration consisted of performing an analysis of data on activity progress, monitoring the processes at the various sites and observing the professionals and their daily tasks at their workplace.

At the same time, the current model of the relationship between user and professional, and between professionals within the system itself, was evaluated by process mapping of all interaction casuistic.

*3. Training healthcare teams in the methodology*

Following the project presentation session and in parallel to the evaluation of the starting point, experts in Lean Management provided training sessions on processes.

In order for the training to reach all healthcare teams and systems, it had previously been suggested to establish internal trainers, which in the medium term may lead to an internal support structure to reach all of Catalonia and ensure the continuity of the model and project results over time. This training was aimed at PCTs and a group of professionals from CatSalut itself.

The training dealt with the Lean, Agile, and Scrum tools and had two goals: (1) to provide the teams knowledge for understanding the new process management and new way of solving problems and evaluating the starting point to define a new working standard; (2) to provide the teams tools for implementing a new coordinated way of working as a team, prioritizing value tasks, or removing bottlenecks.

#### PHASE 2 – Process map and identification of opportunities

The second phase consisted of:

*4. Designing the map of key processes and selecting improvement projects*

In order to conduct an in-depth assessment of the preliminary situation, the current design of primary care processes needed to be considered. With the different process teams, the patient’s process map (VSM) was performed from the first interaction with primary care (requesting an appointment) through the entire care process and up to the discharge or referral to other healthcare systems. VSMs reflect the key processes, a number of pathways and roles of all professionals who interacted with the patient/citizen, and all support tools related to this user’s pathway along the care process.

VSMs were created with the process team appointed at the beginning of the project, given that the professionals were those who had knowledge about the day-to-day tasks and management. Although VSMs may appear similar at all PCTs in Catalonia, it was considered that evaluating the process from the outset with all PCTs was important, as this allowed the team to have a global view of the process, to get involved in the project, and to identify the individual characteristics and opportunities for improvement at each site.

*5. Identifying major improvement actions*

Based on the VSMs that were co-designed with the professionals, an effort-impact matrix was created to prioritize the identified improvement opportunities based on effort and impact in terms of results, and it was decided which of these proposals should be managed in the short term and which should be managed in the medium term. From there, a set of improvements to be implemented in each PCT was suggested.

This proposal for prioritizing the implementation of improvement actions took into account several aspects: (1) impact in terms of the results obtained through the investment to be made, both financially and in terms of time management of the professionals who executed it; (2) scope: suggestions for improvement were classified on the basis of whether they had a local impact (shortest-term implementation) or whether they may be scalable to other PCTs (they were considered cross-sectional and therefore, required a broader approach); and (3) involvement of third parties: the need to include professionals from other healthcare areas was taken into account.

#### PHASE 3 – Deployment of projects - Pilot

*6. Creating working groups to implement the improvement projects*

Phase 3 consisted of the operativization of improvement actions based on the redesign of the processes.

Improvement groups were established within the PCTs to implement and pilot prioritized opportunities in the previous phase and various procedures were followed depending on the scope of the improvement actions.

In order for the working groups to be able to define the plan for implementing the improvement actions, methodological resources were provided to the professionals of each PCT so that they could internally implement the proposals for change with the support of the process engineering team. Each improvement action was evaluated, the new standard was suggested, the action plan was defined in order to conduct the first pilot tests, and follow-up indicators were suggested. All of this was documented using the A3 tool, which helped dissemination among professionals and follow-up of the various improvement projects.

On the other hand, regarding improvement actions classified as potentially scalable to the rest of PCTs, these were recorded by the Support and Monitoring Committee and transferred to the managers of cross-sectional groups so as to evaluate their inclusion in the joint deployment.

#### PHASE 4 – Follow-up proposal

The fourth phase consisted of:

*7. Follow-up of the resulting actions and projects*

For assessing the impact of global implementation, the **Hoshin Kanri** method (Barnabè & Giorgino, 2017; Sales-Coll *et al*., 2021; Moraros *et al*., 2016) was suggested as a method for monitoring indicators and aligning strategic goals, clustered by dimension. Dimensions are areas of action where improvement actions have an impact (e.g., a usual dimension in healthcare is suitability of the clinical practice; within this dimension, indicators that allow a data-driven follow-up of clinical practice suitability are defined. If indicators improve, it means that the implemented improvement actions are having a positive impact on this particular dimension). In order to assess the impact of the improvement projects, it was essential to define those indicators that allowed monitoring the various projects that had been implemented. These indicators were clustered according to the dimensions associated with the projects.

## Results

The scope of the project was large, as it involved reaching all PCTs within the Catalan region (374 PCTs working at 437 primary care centres across 9 healthcare regions) within a 2-year time frame (2021–2022). The deployment of the proposed model was both ambitious and comprehensive. The results detailed below illustrate how this model was systematically implemented, ensuring its equitable integration across the entire region.

### Work team

The unit proposed for the deployment was the PCTs because of being a well-established, well-distributed structure across the region with a defined direction.

In order to reach all PCTs in Catalonia within 2 years (which included juggling sessions with interruptions due to the impact of several COVID-19 waves), it was suggested to set up process teams consisting of at least three PCTs from the same healthcare region, in the same session cycle. After clustering 3 PCTs, a process team consisting of a total of 9 to 12 professionals was created.

PCTs participated in a multidisciplinary team made up of three professionals from the following disciplines: medicine, nursing, and administration.

The roles within the team for each professional were as follows:

- Medical and nursing role – Knowledge about the patient’s care process.
- Administration role – Knowledge about the administration process and communication pathways with the primary care user. Managers and parties who are aware of all procedures and user requests.

In addition to the three main professionals of the PCT, the process team consisted of one or two management professionals and the process engineer, who had the following roles:

- Directive role – Leaders of process teams and facilitators at points blocking improvement actions. Motivating the other colleagues from the PCTs.
- Process engineer role – Methodologist, internal consultant, deployment support, facilitator for improvement groups, and long-term contact for support in the follow-up and maintenance of project indicators.

This results in a total of approximately 1,800 professionals involved in the deployment of the PEiTAPiC, including PCT professionals, process office engineers, external consultants and trainers, as well as experts from the cross-sectional groups.

### Governance

The **Support and Monitoring Committee** held weekly meetings, where the regional team reported the status of the corresponding process team projects, risks, improvement suggestions and information regarding the follow-up, evaluation, and control of CatSalut’s Management Committee, in order to adopt strategic decisions and be aware of the relevant information regarding follow-up and evaluation.

The **Strategic Committee** met once a month and evaluated the project suggestions and what impact they had on each of the defined areas. The improvement relating to the deployed projects was also monitored. These meetings were held using the Hoshin Kanri panel created to do a follow-up of the improvement projects framed in the Dimensions: Reception and Accessibility, Resolution and Continuum of Care, dimensions that had previously been selected with the process teams.

### Deployment

The four phases of the suggested deployment model initially consisted of 8 working days with 2–4 hours each, and each of the process teams were created. In practical terms, to conduct a consistent deployment across the whole region and to achieve this within 2 years, it was decided to conduct a deployment by cycles, consisting of six cycles with a duration of 8 weeks each (a total of 48 cycles).

**Phase 1** included three working days: the first day was for presenting the project (kick-off) and first contact and conversation with the professionals who made up the process team to gain insights into their daily challenges, and the remaining two days were for Lean training. **Phase 2** consisted of two working days, one to perform the VSM and one to prioritize opportunities for improvement using the effort-impact matrix and perform the segmentation of projects between local and cross-sectional groups. Subsequently, **Phase 3** included two working days to establish the improvement groups for each of the selected proposals and evaluate how to conduct a pilot test to check the impact of the actions on the process. Finally, **Phase 4** consisted of one session to follow up the initial results of the pilot tests and extract the final indicators from the implementation period (see the annexes for a more detailed description of phase deployment) and to establish a follow-up procedure (indicators were defined with the process teams for each improvement project).

As the deployment model was implemented, several significant outcomes emerged. A key achievement was the definition of a unified Value Stream Mapping (VSM) model for the Primary Care process, structured into three critical phases: Reception and Accessibility, Care Process, and Resolution. This approach revealed a clear consensus among the teams on the most critical points in patient care, those that frequently posed challenges in daily operations. The first phase, Reception and Accessibility, focused on how users initially interact with the system, including the type of response they receive, and the professionals involved. The Care Process phase encompassed all actions taken while the patient is in primary care, ranging from one-time visits to ongoing management of chronic conditions. The final phase, Resolution, involved the actions requiring coordination with other healthcare services, such as hospitals, which often face challenges due to gaps in coordination.

Similarly, within each phase of the process, consistent patterns emerged in the problems and opportunities for improvement identified by different PCTs. This alignment enabled the definition of 27 transversal improvement project models, addressing these common challenges across the system.

In the end, the deployment led to the development of 721 improvement projects, each addressing these shared challenges and consolidated into the 27 A3-format projects, representing focused, actionable plans designed to drive substantial improvements.

## Discussion

This section provides an in-depth analysis of the results from the deployment of the PEiTAPiC, with a focus on how Lean Methodology has been effectively integrated into the Catalan healthcare system and providing insight into the strengths and weaknesses of the implementation of the defined Work team, Governance and Deployment.

### Methodology

This project contributes to the little existing evidence in the literature (Bruno, 2017; Sisler *et al*., 2017) regarding how the use of the Lean approach, deployed and implemented within the healthcare sector, and more specifically in primary care (Grove *et al*., 2010; Hung *et al*., 2021; Hung *et al*., 2015), can be useful at a professional level, as it promotes organizational changes and leads to lasting improvements through the development of a sustainable work culture.

For the implementation of the Lean methodology to be successful and sustainable over time, it must fulfil several aspects: commitment by management, training, employee participation, and empowerment for the implementation of the suggested changes (Netland, 2015). In addition, two aspects must be considered: the organizational culture and the adoption of appropriate practices (Bortolotti *et al*., 2015). Quite often, implementation is limited to the introduction of tools, and transformation is not sustainable over time (Akmal *et al*., 2020; Parkhi, 2019; Poksinska *et al*., 2017), which means that there is no adherence to the new processes and protocols. Taking all these aspects into account, the strategy followed for the deployment of the project was considered. From the outset, one of the key points in the project was to get all parties involved to understand and learn the methodology until being familiar with it, and this way, to change the working procedures within the care process, working as a team and promoting continuous improvement.

When projects of this magnitude are conducted, both the deployment methodology and the deployment management are important. In the case of primary care, given the significant dispersion and atomization of the management structures, it was very important for the deployment to be consistent across all regions. In order to reach all sites across all regions, it was key to develop cycles, to cluster PCTs for training sessions, to standardize the sessions, and to hold regular Committee meetings, which would allow to do a follow-up and have a cross-sectional and aligned vision towards a common goal.

Managing change in a healthcare environment requires the participation of all healthcare and support personnel involved in the processes to be improved (Ben-Tovim *et al*., 2007; Sánchez *et al*., 2018; Sirvent *et al*., 2016). Therefore, this project was intended to empower primary care to lead and be at the heart of the entire Catalan ambulatory process, as well as to empower all decision-makers to be resolute at all levels. In order to achieve this goal, the focus was on creating and consolidating the teams, both healthcare and support teams.

### Work team

#### Health professionals from the PCTs

The successful management of change in a healthcare environment requires the participation of all healthcare and support personnel involved in the processes to be improved (Coughlan & Coghlan, 2002; Henrique *et al*., 2016; Sales & De Castro, 2021). For this reason, it was important to define which professional roles may be part of the process teams, considering those that could provide a global view of the processes, could promote change in each of the primary care centres, and jointly could form multidisciplinary teams.

#### Support professionals

A key element for a process improvement project with such scope is the creation of a stable team in CatSalut itself so as to involve the management of CatSalut in the deployment so as to drive change from within the organization. This team was established to provide a methodology and support the follow-up once external consultants had finished their role within the project. On the other hand, the Governance Committees allowed the deployment of improvement projects to be aligned with CatSalut’s strategy (Strategic Committee) and following up the progress of projects across the region (Support and Monitoring Committee).

#### Standardized training

Training was suggested for two different reasons. On the one hand, the various deployment sessions introduced training sessions for Lean methodology so that PCT professionals could understand and implement the necessary tools during the deployment of the projects. On the other hand, the management and methodology of the sessions was standardized so that all process engineers could become familiar with the management methodology during the sessions with the first PCTs and subsequently implement the same management with the rest of PCTs across the region.

### Governance

The governance model established for the deployment of the PEiTAPiC framework played a critical role in ensuring the project’s success. By structuring the governance around two committees – the Strategic Committee and the Support and Monitoring Committee – we were able to create a robust framework for both strategic oversight and operational management.

One of the key strengths of this governance model was its ability to foster a strong sense of ownership and collaboration across different areas within the CatSalut. By involving key stakeholders from various sectors in the decision-making process, the project was able to gain widespread support and recognition as a system-wide initiative, rather than being perceived as an isolated effort by the process office. This broad-based engagement was crucial in ensuring that the project received the necessary resources, attention, and commitment from all involved parties, thereby enhancing its impact and sustainability.

However, the reliance on frequent meetings, particularly within the Support and Monitoring Committee, posed challenges in terms of time commitment and resource allocation. Additionally, while the structured approach provided clarity and direction, it also required a high level of coordination and communication, which could be demanding for the teams involved. Despite these challenges, the overall effectiveness of the Hoshin Kanri in driving the project forward and ensuring its alignment with CatSalut’s strategic objectives underscores its value as a critical component of the Lean Methodology implementation.

### Deployment

In order to bring about organizational changes, it was necessary that the processes focused on reducing variability and standardizing primary care flows within the care process for healthcare system users, information flow, and internal communication, so as to organize the day-to-day workload of professionals and identify areas for improvement. Generally, meeting this challenge involves introducing changes in the professionals’ way of working, seeking the best formula for displaying value tasks and prioritizing these tasks based on management as a team. To this end, the introduction of new working methodologies that may ensure a transformational change within primary care was suggested in order to be efficient not only as individual sites but also as components of the network for the provision of local services within the Catalan healthcare system as a whole, and it is here that the Lean and Agile tools have brought great value to this project. To meet the needs of both PCT professionals and users, the starting point had to be the identification of the things they value, which was achieved through training sessions, the involvement of various teams and an assessment of the initial status of primary care across the Catalan region by monitoring, dialoguing professionals, and performing VSMs with process teams. This phase allowed to identify not only value but also the non-value actions needed for the process to be successful and also the opportunities for improvement that impacted on a proposal for change or an improved management of the care process, information channels, communication tools, and logistic flows of the required material and equipment. The entirety of this first analysis phase was the key element in the deployment methodology to subsequently be able to define suitable improvement projects, define the implementation plan, and perform pilots until achieving the improvement of indicators (monitored by the Hoshin Kanri panels). The follow-up of indicators has allowed cycles of continuous improvement and consolidation of those improvements (applying the Deming cycle (Nicolay *et al*., 2012; Zidel, 2006), which are one of the pillars of the Lean approach.

Another tool that allowed a consistent deployment and unification of the new standards was the creation of a digital platform where the various A3-format projects were uploaded and followed up by providing evidence to the sites that wanted to conduct the deployment in the future, the methodology for creating the standard, and the results it yielded. As a result, a set of improvement projects were attained, defined so that they were sustainable over time and significantly contributed to improving activity and efficiency indicators of primary care resources within the Catalan region. The teams that started the first Lean projects have maintained the routine of continuous improvement and have evolved the model for primary care process, achieving a Lean transformation at all levels and putting the patient always first.

Maintaining this routine of long-term continuous improvement will help to ensure patient safety and care quality, optimize process resources, reduce waiting times, and increase the satisfaction of professionals and users (Bonome *et al*., 2016; Joosten *et al*., 2009; de Souza *et al*., 2021). These factors are essential for primary care to be cost-effective, decisive, and high quality in the long term within the healthcare systems.

### Limitations

It must be noted that a project of this magnitude also has a number of limitations. First, although we worked with up to more than 1800 professionals, primary care employs around 30 000 professionals and therefore, there is a long way to go for all professionals to learn and be familiar with the new working methodologies. Second, the acceptance of a transformation process such as the one that has been suggested, although it was approached as an inclusion of professionals and long-term implementation, has been unequal at the various sites across the region (some have shown less resistance to change than others). Third, the duration of the project and the delivery of results largely depend on a long-term vision and therefore, although significant results have been attained, a large-scale operational improvement will still be slow.

Finally, it is worth noting that aligning a body of professionals with differing interests, such as primary care professionals, CatSalut organizations, or the Catalan Ministry of Health, may be challenging and may require a significant communication effort to promote the benefits of such a project at all levels.

## Conclusions

In conclusion, the successful implementation and deployment of the PEiTAPiC model across Catalonia has provided several key insights into the factors critical to the project’s success. The following points summarize the essential elements that have emerged from this extensive deployment. These conclusions not only reflect the strengths and limitations of the methodology used but also offer a strategic framework that can be replicated in other regions for similar initiatives.

- Such a project is only possible with leadership and managerial alignment.
- A well-defined strategy is essential to execute a consistent deployment across the region in order to achieve a critical number of professionals motivated to achieve improvement. Such strategy may include cycles, iterations, and standardization of the deployment sessions.
- The involvement of front-line professionals in the identification and implementation of improvement projects ensures the success of the deployment strategy.
- The training has been key for professionals to understand the methodology and tools used to provide them with autonomy for the deployment of subsequent projects, thus promoting continuous improvement.
- Communication of the project’s goals and the improvements expected at all levels has prevented resistances from hindering the development of the project.
- This is a long-term project, so we cannot expect to obtain significant results quickly.
- A standard for the deployment of the primary care improvement project has been created at a strategic level. This standard may be replicated in other regions.

## Figures and Tables

**Figure A1.**
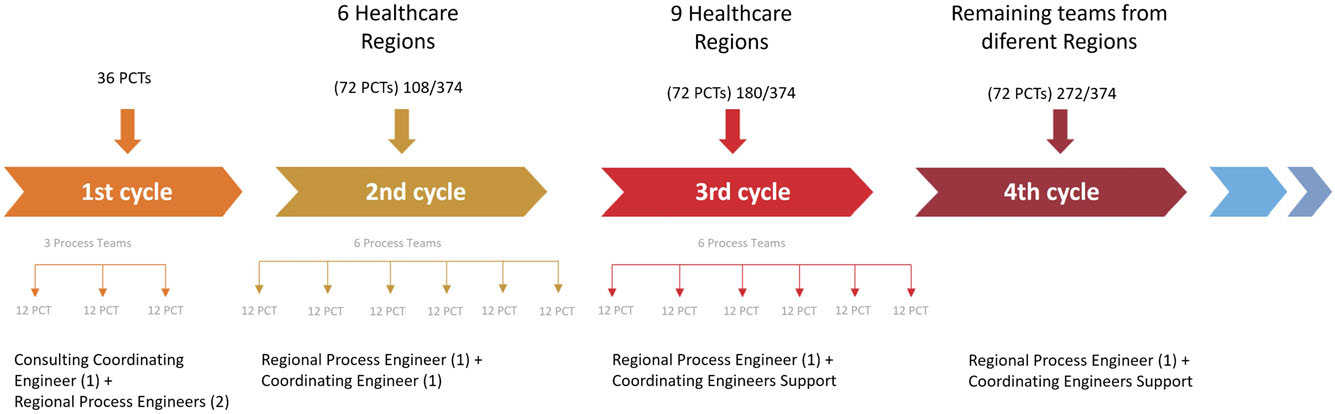
Model followed for the deployment by cycles.

**Figure A2.**
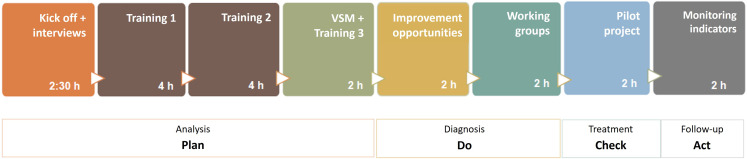
Structure and duration of the 8 working days that make up a cycle of the deployment plan.

## Appendix

### Deployment of the phases

Starting with the clustering of process teams and due to its relevance for consistent deployment across the region, thus creating a critical mass, a deployment by cycles was adopted (Figure A1).

In the early approach, in each cycle, there were eight working days where all stages of the four model’s phases were implemented. One session per week with a duration of 2 hours was suggested, with the exception of the kick-off and the training sessions, with a duration of 2.5 and 4 hours, respectively (see Figure A2).

The deployment was conducted in a similar way for all cycles and in the following order, despite minor variations in the number of sessions. Adding two more days of practical training for the development of local projects from the second cycle. According to the proposed initial schedule, since each cycle consisted of 8 weeks and considering that there were additional free weeks for potential date changes of the working sessions, project review, and documentation, the deployment required a minimum of 46 weeks from the second cycle.
